# Testing Consultation Recordings in a Clinical Setting With the SecondEars Smartphone App: Mixed Methods Implementation Study

**DOI:** 10.2196/15593

**Published:** 2020-01-21

**Authors:** Amelia Hyatt, Ruby Lipson-Smith, Bryce Morkunas, Meinir Krishnasamy, Michael Jefford, Kathryn Baxter, Karla Gough, Declan Murphy, Allison Drosdowsky, Jo Phipps-Nelson, Fiona White, Alan White, Lesley Serong, Geraldine McDonald, Donna Milne

**Affiliations:** 1 Cancer Experiences Research Peter MacCallum Cancer Centre Melbourne Australia; 2 Department of Nursing University of Melbourne Melbourne Australia; 3 Victorian Comprehensive Cancer Centre Melbourne Australia; 4 Centre for Cancer Research The University of Melbourne Melbourne Australia; 5 Sir Peter MacCallum Department of Oncology University of Melbourne Melbourne Australia; 6 Health Information Management Peter MacCallum Cancer Centre Melbourne Australia; 7 Prevention and Wellbeing Peter MacCallum Cancer Centre Melbourne Australia

**Keywords:** mHealth, cancer, mobile apps, implementation, pilot, consultation audio recording

## Abstract

**Background:**

Health care systems are increasingly looking to mobile device technologies (mobile health) to improve patient experience and health outcomes. SecondEars is a smartphone app designed to allow patients to audio-record medical consultations to improve recall, understanding, and health care self-management. Novel health interventions such as SecondEars often fail to be implemented post pilot-testing owing to inadequate user experience (UX) assessment, a key component of a comprehensive implementation strategy.

**Objective:**

This study aimed to pilot the SecondEars app within an active clinical setting to identify factors necessary for optimal implementation. Objectives were to (1) investigate patient UX and acceptability, utility, and satisfaction with the SecondEars app, and (2) understand health professional perspectives on issues, solutions, and strategies for effective implementation of SecondEars.

**Methods:**

A mixed methods implementation study was employed. Patients were invited to test the app to record consultations with participating oncology health professionals. Follow-up interviews were conducted with all participating patients (or carers) and health professionals, regarding uptake and extent of app use. Responses to the Mobile App Rating Scale (MARS) were also collected. Interviews were analyzed using interpretive descriptive methodology; all quantitative data were analyzed descriptively.

**Results:**

A total of 24 patients used SecondEars to record consultations with 10 multidisciplinary health professionals. In all, 22 of these patients used SecondEars to listen to all or part of the recording, either alone or with family. All 100% of patient participants reported in the MARS that they would use SecondEars again and recommend it to others. A total of 3 themes were identified from the patient interviews relating to the UX of SecondEars: empowerment, facilitating support in cancer care, and usability. Further, 5 themes were identified from the health professional interviews relating to implementation of SecondEars: changing hospital culture, mitigating medico-legal concerns, improving patient care, communication, and practical implementation solutions.

**Conclusions:**

Data collected during pilot testing regarding recording use, UX, and health professional and patient perspectives will be important for designing an effective implementation strategy for SecondEars. Those testing the app found it useful and felt that it could facilitate the benefits of consultation recordings, along with providing patient empowerment and support. Potential issues regarding implementation were discussed, and solutions were generated.

**Trial Registration:**

Australia and New Zealand Clinical Trials Registry ACTRN12618000730202; https://www.anzctr.org.au/Trial/Registration/TrialReview.aspx?id=373915&isClinicalTrial=False

## Introduction

### Using Mobile Health to Strengthen Patient-Centered Care

Mobile health (mHealth) describes health care facilitation or delivery via mobile devices such as smartphones or tablets [1]. Hospitals are increasingly looking to mHealth and electronic health (eHealth) innovations to improve safety and quality [2-4]. Benefits can include low-cost health system integration and improved engagement, communication, and care delivery, particularly for low income and other disadvantaged patient populations [1,3,5,6]. In addition, patients are increasingly becoming interested in accessing technological solutions designed to empower them to effectively and independently manage their care [7]. Furthermore, the increasing availability of smartphones has allowed for the development of mHealth interventions to reduce inequities in health care service delivery and barriers such as poor health literacy [5].

### Consultation Audio-Recordings

Consultation audio-recordings are one such technological solution. Patients report difficulty remembering and understanding information in the health care context, with these difficulties exacerbated by shock or stress [8]. Research has demonstrated that providing recordings of medical consultations can be a useful tool to combat this. With the help of recordings, patients’ report improved understanding and recall of key medical information and improved engagement and satisfaction with their doctor and health service [9-15]. Previous consultation-recording research using technologies such as Dictaphones or cassette tapes identified numerous potential barriers to health service implementation including lack of sustainability, data security, poor sound quality, and high clinical burden [13,14,16].

### The SecondEars App

The SecondEars smartphone app was developed by our team as an evidence-based mHealth solution to overcome barriers described, and to facilitate implementation of consultation audio-recordings into routine clinical care [17]. An experience-based co-design methodology [18] was used to develop the app, with patients, doctors, nurses, health information management, and information technology representatives involved in all facets of the project from concept creation to design and development [17].

### The Need for Pilot Testing and User Experience

Nevertheless, even evidence-based mHealth technologies such as SecondEars are not easily integrated into everyday clinical care. Most mHealth interventions fail to be implemented after pilot-testing [19,20] with the term *pilotitis* created to describe this phenomenon [21,22]. This is likely due to a number of reasons. It is well documented that the foremost barrier to acceptance and use of a new technology is poor attention to user experience (UX), or *usability* [23], and pilot trials have typically done a poor job of collecting the information needed to remedy usability problems before implementation [20]. UX in this context refers to the dynamic, context-dependent, and subjective singular and accumulated experiences a user has as a consequence of interaction with a technology [24]. Positive UX and subsequent user decisions regarding acceptability, satisfaction, and feedback regarding the intervention have been found to be directly linked to whether an innovation will be adopted by its target population [4,23,25].

mHealth apps are also often not piloted in the actual environment where they will be used [20], meaning that barriers to feasibility or use in *real-world* settings cannot be adequately identified. Implementation and system development issues need to be identified and understood as part of comprehensive pilot testing [3,26,27]. mHealth innovations tend to be more complex than other technologies as they often require integration with multiple existing systems and must be appropriate for a variety of users [21]. Effective communication between stakeholders regarding application function, underlying public health improvement purpose, and function within the health system is also optimal for implementation [28]. Many unsuccessful pilot projects have in common a lack of consideration of this complexity, poor stakeholder engagement, and a failure to investigate and collect the requisite data regarding key elements essential for sustainable and scalable system implementation [21,29].

### Objectives

This study aimed to pilot the SecondEars app within a real-world clinical setting to identify factors to optimize implementation. As identified in the literature, the following elements were deemed essential for consideration before implementation:

1. Patient UX and subsequent decisions regarding acceptability, utility, and satisfaction of SecondEars app
2. Health professional perspectives about issues, solutions, and strategies for effective implementation of SecondEars.

## Methods

### About the SecondEars App

SecondEars was designed so that patients can record conversations during appointments they think will be most helpful and with whichever health professional they wish (providing the health professional gives their permission). Appointments may include (but are not limited to): one-on-one doctor consults, nurse-led treatment or symptom education sessions, and appointments with allied health professionals. SecondEars also includes features (see Multimedia Appendix 1) which allow patients to write and associate notes with each recording, such as questions for their next appointment. Each recording can be labeled, or *tagged*, according to a particular health professional, the consultation type, or any other association the patient wishes to make, for example, diagnosis information and treatment plan.

### Data Management and Security

Audio files made by patients can be uploaded from the SecondEars app and stored within a secure cloud server prior to playback. This storage facility was designed so that the audio-recordings can be accessed by the health service’s patient health information services and information technology teams. Once uploaded, patients can share their audio files via the SecondEars app using any of the standard services on their smartphone (eg, email and messaging services with the exclusion of social media apps such as Facebook).

### Version Details

Version 1.0 of SecondEars was pilot tested in this study. This version was only available through the Apple testing service TestFlight. Version 1.0 was operated by all participants as no revisions or updates were implemented during the study period.

### Theoretical Framework

The Consolidated Framework for Implementation Research (CFIR) was chosen as the theoretical framework for this study. The CFIR is a taxonomy of health implementation theories and the associated constructs that have been shown to be effective for facilitating successful implementation of health innovations [30]. The following CFIR domains were used to inform development of study processes: (1) the characteristics of the intervention being implemented, (2) characteristics about individuals who are involved in the implementation process, (3) inner setting refers to the features of the context where the technology is to be implemented, that is, hospital, and 4) the process by which implementation is undertaken [30].

### Pilot Testing, User Experience, and Implementation Data Collection

A mixed methods implementation study was employed to collect data regarding the feasibility and UX of the SecondEars consultation audio-recording app in a clinical setting. Combining qualitative and quantitative data is recognized as the optimal approach to describe and understand user perceptions of mHealth interventions for cancer patients’ self-management [4]. Data were collected concurrently, with the greatest weighting on the qualitative data, in line with the study’s focus on description and exploration.

The study was conducted at the Peter MacCallum Cancer Centre (Peter Mac) in Melbourne, Australia. Written informed consent was obtained from all study participants, and the study protocol was approved by the Peter MacCallum Cancer Centre Human Research Ethics Committee (study number: 16/07L). This study was registered with the Australian and New Zealand Clinical Trials Registry (ACTRN12618000730202).

### Participants

#### Patients and Family

Patients with a scheduled out-patient consultation at Peter Mac were invited to test the SecondEars app by downloading and using it to audio-record a consultation with a participating health professional. To be eligible, patients needed to be aged ≥18 years; able to read, write, and speak English; and have access to and ability to operate an iPhone or iPad.

#### Clinical Staff

Oncologists, nursing, and allied health staff working in the following departments were invited to take part: Nutrition, Physiotherapy, Speech Pathology, Skin and Melanoma, Urology, and Lung outpatient clinics. All staff in these departments were made aware of the study via presentations at staff meetings and email circulations. Staff who expressed interest were then invited to participate via email.

### Measures

#### Patient Demographic Questionnaire

A customized, self-report measure was used to gather patient demographic, appointment, and technological ability characteristics comprising age, postcode, sex, consultation information, and self-reported skill with smartphone technology.

#### Patient Mobile App Rating Scale

The Mobile App Rating Scale (MARS) was used to collect data regarding the CFIR construct: intervention (app) characteristics [30,31]. The MARS uses a 5-point scale to assess app quality (from 1=inadequate to 5=excellent). Researchers can select to use only the categories of the MARS that are relevant to their application; this study used aesthetics (3 items), functionality (4 items), and subjective app quality (4 items) [31].

#### Patient and Health Professional Interviews

Semistructured interviews were used to collect data about the UX of the SecondEars app in a clinical setting (patients and health professionals) and at home (patients only), and perspectives regarding implementation and stakeholder engagement (health professionals only). Patient interviews were designed to elicit responses regarding user perspectives and experiences of each feature and function of the app, and how the app was integrated into their overall health care experience. Health professional interviews were designed to investigate perspectives on implementation barriers, facilitators, and strategies for effective implementation. Most questions in both interview schedules were open-ended, but some were closed to provide quantifiable data about app use (eg, *How many times have you listened to the audio-recording?*).

### Procedures

#### Pilot Testing

Health professionals were informed of the pilot study at multidisciplinary team meetings. Those who volunteered to take part in the study provided consent to have consultations audio-recorded and complete an interview. Patients scheduled to attend outpatient appointments with consenting health professionals were screened for eligibility between February 19 and July 31, 2018. Eligible patients were approached via telephone before their appointment; those interested were emailed an information sheet and consent form and instructions on how to download the SecondEars app using TestFlight. TestFlight is part of the iOS development program, which allows users to test mobile apps before they are listed on the Apple App store. A member of the research team met the patient in the waiting room before their consultation to collect the signed consent form, provide assistance with downloading and setting up the app if required, and collect demographic information. If any family or friends were attending the appointment with the patient, they were also given information about the study and asked to provide consent to the consultation being audio-recorded. App use was not directed by the researchers during or after the consultation; it was up to the patient participants to use the app as they chose.

#### Interviews

One week after their recorded consultation, patient participants completed an interview and the MARS via telephone. A copy of the MARS was emailed to each patient participant before the interview, and responses were provided verbally by participants, at the end of the interview. Some participants had chosen to ask a family member to manage the app for them. These participants had the option of nominating their family member to participate in the interview with them, or on their behalf. Participating health professionals completed a face-to-face, audio-recorded interview with a researcher at the conclusion of the study.

### Data Analysis

#### Quantitative Data

The MARS was scored using published guidelines [31]. Responses to items from the MARS aesthetics and functionality categories were averaged separately to provide subscale scores.

Descriptive statistics were used to summarize data collected using the customized, self-report survey, the MARS and closed-ended interview questions. Nominal data were summarized using counts and percentages. Continuous data were summarized using means and standard deviations.

#### Qualitative Data

Patient, carer, and health professional interviews were analyzed using interpretive description methodology, which is designed to be used in addressing questions of clinical utility in health care [32,33]. Transcribed interview data were sorted into codes, then categories and themes using QSR International’s NVivo 11 software [34]. A total of 2 members of the research team analyzed the data. RLS analyzed the patient interviews, and AH analyzed the health professional interviews. Group discussions were held with the project team to review the categories and themes. If there were differences in opinions, discussion would continue until a consensus was reached regarding interpretation of the data [35].

## Results

### Pilot Testing Sample

#### Patients

Of the 51 patients who were eligible for the study, 30 consented to participate (59% consent rate). Of those who declined, a majority (n=12) were not interested in participating in research, 2 were not confident with technology, 2 were scheduled to have their appointment via telehealth, 2 did not want any distractions from their consultation, and 3 listed personal reasons (eg death in family and having nurse as partner).

#### Recorded Their Consultation

Of the 30 patients who consented to participate, 6 did not record their consultation. In all, 3 had their appointment changed or cancelled, 2 were unable to download the app as the hospital WIFI was not working or they had forgotten their Apple ID password, and 1 forgot to press record. The total number of patient participants included in the study was therefore 24 (see Table 1).

#### Health Professionals

A total of 18 health professionals volunteered to take part in the trial; however, 8 of these did not have any eligible patients agree to take part. Therefore, the total number of health professionals included in the study was 10 (see Table 1).

**Table 1:** Participant demographic information (N=24).

Characteristics			Value
**Patients**			
	Age (years), mean (SD, range)		58 (10, 39-75)
	**Sex,** **n** **(%)**		
		Male	17 (71)
		Female	7 (29)
	**Cancer type,** **n** **(%)**		
		Lower gastrointestinal	5 (21)
		Lung	3 (13)
		Melanoma	7 (29)
		Urology	8 (33)
		Head and neck	1 (4)
	**Consultation type,** **n** **(%)**		
		Surgical oncologist	6 (25)
		Medical oncologist	15 (63)
		Oncology nurse and physiotherapist (joint consult)	2 (8)
		Speech pathologist	1 (4)
	**Tech ability,** **n** **(%)**		
		Beginner	2 (8)
		Intermediate	17 (71)
		Advanced	5 (21)
	**Device used, n (%)**		
		iPhone (own)	15 (63)
		iPhone (partner’s or family’s)	7 (29)
		iPad	2 (8)
**Health professionals (n=10)**			
	**Sex, n (%)**		
		Male	5 (50)
		Female	5 (50)
	**Specialty, n (%)**		
		Surgical oncologist	1 (10)
		Medical oncologist	5 (50)
		Oncology nurse	1 (10)
		Physiotherapist	1 (10)
		Speech pathologist	2 (20)

#### Interviews

Of the 24 participants who recorded their consultation, 21 patients and 4 carers completed the interview (2 carers completed the interview on behalf of the participant, 2 carers completed an interview in addition to the participant, and 1 participant was lost to follow-up). One health professional did not complete a follow-up interview due to extended leave; therefore, a total of 9 health professionals completed the interview. Closed-ended interview data are summarized in Table 2.

**Table 2:** Participant and family use of the SecondEars app

App and recording use		Patients (n=21)^a^, n (%)	Carers (n=4), n (%)
**Number of participants who listened to all or part of audio-recording**			
	All	11 (56)	3 (75)
	Partial	8 (36)	1 (25)
	Did not listen	2 (8)	0 (0)
**When listening to the audio-recording, was anybody else present^b^**			
	No, listened to it alone	9 (39)	1 (25)
	Yes, listened with a spouse/partner	11 (48)	3 (75)
	Yes, listened with another family member	3 (13)	0 (0)
**Number of participants who used the share function**			
	Used function to share recording	4 (19)	2 (50)
	Intended to use function to share recording	2 (10)	0 (0)
	Did not use share function	15 (71)	2 (50)
**The audio-recording was shared with**			
	Self	2 (50)	1 (50)
	Child	1 (25)	1 (50)
	Partner	1 (25)	0 (0)
**Intended to share with**			
	Child	1 (50.0)	0 (0)
	General practitioner	1 (50.0)	0 (0)
**Number of participants who used the notes function**			
	Did use	2 (10)	0 (0)
	Did not use	19 (90)	4 (100)
**Number of participants who used the labeling function**			
	Did use	6 (29)	2 (50)
	Did not use	15 (71)	2 (50)

^a^Including two patients that completed only the interview, not the Mobile App Rating Scale.

^b^Does not add up to 19 for patients because some people listened to it with more than one person.

### User Experience and Acceptability, Utility, and Satisfaction

#### Patient and Carer Qualitative Interviews and the Mobile App Rating Scale Results

**Figure 1:**
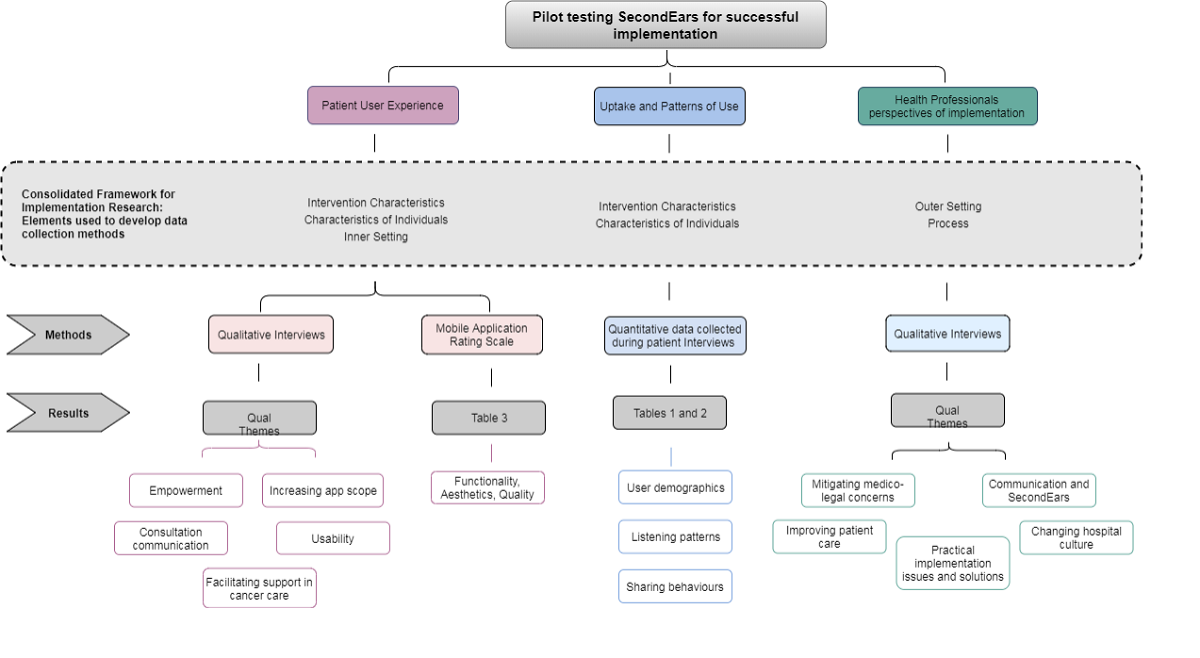
Synthesis of results.

UX interviews were completed with 21 patients, 4 family members, and 9 health professionals. Quantitative app utility data collected via the MARS are displayed in Table 3. The themes that emerged from the qualitative interviews with patients and carers are described below. A total of 3 themes summarize the usability, acceptability, utility, and satisfaction that patients and family members identified and described as relevant to their experience testing SecondEars (see Figure 1 for a summary of the results).

#### Empowerment and Reassurance

Patients described the SecondEars app as a *safety net* that helped them to feel secure and in control. Having the app on their own smartphone gave the participants flexibility and choice about how and when to make and listen to the recordings. Participants liked that they could choose to listen to the recording in an environment where they felt comfortable, as this helped to control the emotional aspects associated with relistening to health information, and provided an antidote to rumination. Using SecondEars to confirm that their interpretation or recall was correct gave participants confidence and reassurance—the app was literally at their fingers.

If something sort of goes over your head a little bit during the meeting you’ve got it [the app] there you know and you can actually listen to that audio at any time and it will sort of clear what’s going on in your head... it really does take a load off your mind because you can hear everything back.

Female patient, aged 55, P22

**Table 3:** Results from the Mobile Application Rating Scale (participants, N=23).

Subscale^a^			Value, n (%)
**Functionality^b^**			
	**Performance: How accurately/fast do the app features and components work?**		
		App is broken; no/insufficient/inaccurate response (eg, crashes/bugs)	0 (0)
		Some functions work, but lagging or contains major technical problems	0 (0)
		App works overall. Some technical problems need fixing, or is slow at times	0 (0)
		Mostly functional with minor/negligible problems	6 (26)
		Perfect/timely response; no technical bugs found	17 (74)
	**Ease of use: How easy is it to learn using the app?**		
		No/limited instructions; menu labels, icons are confusing; complicated	0 (0)
		Takes a lot of time or effort	0 (0)
		Takes some time or effort	1 (4)
		Easy to learn (or has clear instructions)	6 (26)
		Able to use app immediately; intuitive; simple (no instructions needed)	16 (70)
	**Navigation: Does moving between screens make sense, all links present?**		
		No logical connection between screens at all/navigation is difficult	0 (0)
		Understandable after a lot of time/effort	1 (4)
		Understandable after some time/effort	2 (9)
		Easy to understand/navigate	4 (17)
		Perfectly logical, easy, clear, and intuitive screen flow throughout and/or shortcuts	15 (64)
	**Gestural design**		
		Completely inconsistent/confusing	0 (0)
		Often inconsistent/confusing	1 (4)
		Okay with some inconsistencies/confusing elements	1 (4)
		Mostly consistent/intuitive with negligible problems	5 (22)
		Perfectly consistent and intuitive	16 (70)
**Aesthetics^c^**			
	**Layout: graphic design, overall visual appeal, color scheme, and stylistic consistency**		
		Very bad design, cluttered, options impossible to select, locate, see, or read	0 (0)
		Bad design, random, unclear, some options difficult to select/locate/see/read	0 (0)
		Satisfactory, few problems with selecting/locating/seeing/reading items	2 (9)
		Mostly clear, able to select/locate/see/read items	5 (22)
		Professional, simple, clear, orderly, logically organized	16 (70)
	**Graphics**		
		Appears amateur, very poor design, disproportionate, stylistically inconsistent	0 (0)
		Low quality/low resolution graphics; low quality visual design—disproportionate	0 (0)
		Moderate quality graphics and visual design (generally consistent in style)	2 (9)
		High quality/resolution and visual design, mostly proportionate, consistent in style	11 (48)
		Very high quality/resolution and visual design, proportionate, consistent in style	10 (43)
	**Visual appeal**		
		Ugly, unpleasant to look at, poorly designed, clashing, mismatched colors	0 (0)
		Bad: poorly designed, bad use of color, visually boring	1 (4)
		OK: average, neither pleasant, nor unpleasant	5 (22)
		Pleasant: seamless graphics, consistent and professionally designed	9 (39)
		Beautiful: very attractive, memorable, stands out; use of color enhances app	7 (30)
**App subjective quality**			
	**Would you recommend this app to people who might benefit from it?**		
		Not at all I would not recommend this app to anyone	0 (0)
		There are very few people I would recommend this app to	0 (0)
		Maybe there are several people whom I would recommend it to	1 (4)
		There are many people I would recommend this app to	2 (9)
		Definitely, I would recommend this app to everyone	20 (87)
	**How many times would you use the app in the next 12 months if it was relevant to you?**		
		None	0 (0)
		1-2	3 (13)
		3-10	9 (39)
		10-50	10 (44)
		>50	1 (4)
	**Would you pay for the app?**		
		No	7 (30)
		Maybe	6 (26)
		Yes	10 (44)
	**What is your overall star rating of the app? (number of stars)^d^**		
		1 (one of the worst apps I have used)	0 (0)
		2	0 (0)
		3 (average)	2 (9)
		4	15 (65)
		5 (one of the best apps I have used)	6 (26)

^a^The Mobile App Rating Scale (MARS) was only offered to patients who recorded their consultation. In addition, 2 patients did not complete the MARS as had delegated the operation of the app to their carer; therefore, their carers completed it instead.

^b^The mean functionality subscale score was 4.6 (SD 0.7).

^c^The mean aesthetics subscale score was 4.3 (SD 0.8).

^d^Overall star rating (mean [SD]) was 4.2 (0.6).

The design of the app allowed participants to skip over sections of the audio-recording that they found less relevant. Some participants took the initiative to test the app before their consultation or to use the app in ways unintended by the app developers, for example, sharing the recording to their own email address so that they could store a back-up copy.

#### Facilitating Support in Cancer Care

Patients felt that SecondEars facilitated support from family and health professionals. Participants often used the app collaboratively, as it made it easier for their family members and friends to be involved in their care. Participants operated the device with help from family, chose to listen to the audio-recording with others, or to share the recording via the share function. One participant mentioned that his wife might use the app to record his future appointments, even though he did not plan to do so. Being able to relisten to the consultation facilitated discussion with family and helped to settle disagreements.

It really satisfied us that we could revisit the conversation we had with [health professional]. For example we would talk to our daughter and she was quite impressed with it too, you know, in 15, 20 minutes you can do an awful lot of talking... we sent [shared recording of health professional appointment via app] through to our daughter in Shepparton and as I said she was quite impressed with it.

Male patient, aged 75, P11

Participants also suggested that SecondEars could be used to share consultation audio-recordings with other members of their treatment team, including health professionals external to the hospital (such as GPs), to keep everyone informed. However, some participants described feeling apprehensive or reluctant to burden others with information by sharing recordings.

#### Ease-of-Use

SecondEars was described as functional and user-friendly, with good sound quality, even when there were multiple people speaking in the room. In one of the testing situations, the recording was made in a lecture theatre education session with many patients and carers attending, with this also reported as having adequate sound quality. Particularly, participants liked the simple design and scope, which they felt would enable access for a wide range of people, including older adults. Almost every participant commented on how simple and easy it was to use SecondEars, even when in a busy and emotional clinical environment.

Very simple just the layout and graphics very simple. Probably very good for people who are not technologically savvy... there’s a big record button in the middle of the screen you just press it and away you go... just about anybody could use without sort of stuffing it up [laughs].

Male patient, aged 64, P04

Most participants reported no technological issues, and they liked the convenience SecondEars provided by enabling them to record and relisten on their own phone. Some participants reported feeling worried that uploading the audio-recording would be time consuming if the internet or mobile reception was poor; however, this was not the case in practice as most participants found recordings easy to upload. Some participants relied on family to use the app for them. Patient participants who mentioned in the interviews that they were frightened of technology were all part of a patient/carer dyad where the carer primarily operated the app. One nontech-savvy participant reported that he did not skip through irrelevant sections of the consultation recording because he was worried about accidentally deleting something. One participant suggested adding some frequently asked questions to the instructions. Others felt it was important to be able to ask a member of staff for help with the app if needed.

Like I said I’m not very adventurous on that [technology]. I wouldn’t do that [tagging] without one of the kids being around.

Female carer for male patient, aged 71, P20a

The *core* functions of the app (audio-recording, uploading, listening, and sharing the audio-recording) were used by most participants, however few used the other functions, such as the labeling feature or the note-taking function. Participants made suggestions to make the app more functional, such as allowing patients to *bookmark* certain sections of the recordings. People also expressed surprise the system had not already been implemented into usual care given how useful and easy they had found SecondEars. Furthermore, 1 participant emphasized the importance of involving clinicians in the implementation process and ensuring the app had full support of the hospital before rolling it out.

#### Changing Communication

Participants reported being conscious of SecondEars during the consultation, but that, on the whole, they did not find it obtrusive. However, 3 participants speculated whether having SecondEars in the consultation may result in clinicians being more cautious or guarded because they know they are being audio-recorded. In all, 2 of these participants felt this may improve the quality of the consultation for the patient, whereas the other was not so sure. One participant thought that being able to use SecondEars on their phone with clinician permission solved the dilemma of having to ask the clinician to record the consultation and having a Dictaphone prominently displayed between them, or with the alternative of covertly recording.

We were all quite conscious of it being there during the consultation but at the same time you know it didn’t distract us from what we were talking about... I mean we were conscious in the sense that we just put it in between us so that we’d both be heard.

Male patient, aged 58, p28

#### Increasing App Scope

A participant (as quoted below) suggested that using the app could help them to be a more informed and engaged health consumer. A total of 2 participants mentioned that they, or a member of their family, could use the audio-recording to take notes after the appointment. Some participants suggested other uses for the app beyond its original scope, such recordings being used for communication skills education and training for clinicians, or as a form of preparation for new patients to learn what to expect. One participant mentioned that the app could be used to play recordings to other health professionals to correct inconsistencies in information provided. Although some participants felt that the app would be most useful at initial diagnosis, others felt they would use it for all their medical consultations at Peter Mac and elsewhere, and with other types of health professionals such as physiotherapists. They felt that SecondEars would be most useful if all clinicians were on board and if the app was available at other health care organizations and for other illnesses besides cancer.

I’m very much process person and um I ask lots of questions um because I believe in being a responsible health consumer and an informed health consumer and it strikes me that your app helps me to do exactly that…um I would say what would I say, I would say look if you want to make an informed choice about your treatment um then this app will help you to do that.

Female patient, aged 67, P27

### Mobile App Rating Scale

All participants rated that they would use the app again, with a large proportion (10/23, 44%) selecting that they would use the app 10 to 50 times (see Table 3 for a summary of the MARS results). When asked whether they would recommend SecondEars to others, 100% of participants stated they would, with the majority confirming that they would definitively recommend to everyone they knew (20/23, 87%). Participants predominantly rated the app highly according to functionality, aesthetics, and subjective quality, with mean subscale scores all greater than 4 (out of a possible 5). A small minority of participants found the app often or sometimes confusing (2/23, 4%); or thought the design was bad (1/23, 4%). Interestingly, 44% (10/23) of participants indicated they would pay to use SecondEars.

### Implementation

#### Health Professional Qualitative Interviews

Although health professionals were not direct users of the app themselves, the app design and purpose means that their involvement and engagement are essential for successful implementation. Health professionals were asked to focus on issues, solutions, and strategies for effective implementation of the SecondEars app. A total of 5 themes emerged from these qualitative interviews.

#### Changing Hospital Culture

All health professional participants supported the idea of SecondEars being made available to all patients, and that it should remain under patient control. Health professionals recognized that not all patients would have the need or capacity to use SecondEars; therefore, patients should self-identify for the service. Maintaining patients’ control over SecondEars also ensures that the app would not incur additional demands on clinical staff.

I absolutely 100% think it [SecondEars] should be part of usual care. End of the day, see more patients, help more people um it’s only a good thing. I think this is this is where health care is going. I think it just needs to be embraced as much as it can be and rolled out.

C1

Many health professionals felt that patients were already leading the way in this area, as requests to use smartphones to record consultations were already common. Patients already realized the benefits of recording important information. Some health professional participants also reported having discovered patients recording appointments covertly in the past, with this seen as further impetus for the health service to embrace the technology.

Patients will sometimes do this anyway... they’ll say: “would you mind awfully if I recorded this?” you know?

C3

Health professionals discussed strategies that could be employed to begin culture change within the hospital. Clinical champions or change agents were seen as being useful in driving group acceptance of new technology for both health professionals and patients. Nurses were suggested as hospital-wide change agents, as they are the largest group of health professionals in the hospital and carry lots of influence with patients and staff alike. Having a SecondEars champion or representative in each clinical service to assist with implementation was also suggested. Likewise, marketing and social campaigns for both health professionals and patients were suggested as good methods to employ to change hospital culture, including: promotions at multidisciplinary team meetings, an internal marketing campaign, with patient stories, and engaging with the communications team for both internal and external media.

I think you need champions for sure and I think if you’re going to roll out you’d need clinical champions and you need patient champions as well.

C4

One doctor likened the potential for mHealth culture change to the ubiquity to which apps are accepted and used by customers in the airline industry. People boarding a plane automatically search for entertainment apps, and so likewise, patients could become accustomed to investigating what mHealth services are available upon arrival at a hospital.

#### Mitigating Medico-Legal Concerns

Health professionals felt that early and open discussion and planning for medico-legal concerns would assist hospital culture change. SecondEars was thought to have the potential to improve transparency and communication, and this could be reflected in discussions about legal issues, and how this is protective for both parties.

Have people... point out [to health professionals] some of the legalities associated with the system and to say that yeah that it’s going to be actually in some ways protecting you rather than it being something that can be um be a risk.

C5

Health professionals briefly discussed the potential for consultation recordings to form part of legal proceedings. However, the concerns about recordings being used in legal settings were predominantly discussed in the context of ‘other health professionals may potentially be worried about this;’ all clinical staff interviewed were comfortable with the process in their own practice and saw only the benefits of recordings for both themselves and patients.

...people’s mind often go to medico legal things whereas to me that’s the least of worries you know. Yeah I mean I’ve been working 15 years I’ve never been you know called up for anything um... you know and... you know ninety nine point nine percent of people do a very good job within their roles so I don’t think necessarily that’s the issue. I think the benefit is more around for us I would see it more for the patient. Getting a better understanding of what their treatment is and being able to share it rather than ah protecting ourselves or covering ourselves.

C10

Health professionals also thought that having a recording of the consultation would prove beneficial in this context, particularly when using something like SecondEars, which also ensures a copy is saved within the hospital medical record.

From a kind of legal reassuring point of view I suppose to know that if the patient is unhappy or thinks you said something that wasn’t true or you know…any of those things that you kind of worry about as a health professional yourself…to know that [the hospital] holds a copy of [the recording]

C1

#### Improving Patient Care

Health professionals also felt that SecondEars could facilitate family involvement in patient care. Echoing the sentiments of patient participants, health professionals also felt that it would be reassuring for both health professionals and patients to know that a copy of all information was saved and could be relistened to. The ability of health professionals to listen to and share recordings among themselves was also seen as valuable for numerous reasons: to reduce duplication of information provided to patients, improve continuity of care, remind themselves of what information they had communicated to patients, and to assist during sudden transfer of care. Patients having recordings was thought to potentially reduce questions about forgotten information, but conversely, also increase patient engagement and discussion about information provided. Several health professionals also mentioned that SecondEars could be beneficial during informed consent procedures for treatment and/or clinical trials.

I absolutely think it would be a big improvement to patient care. It’s something they’d [patients] really value and staff would value too. For me I like to know that they had that information at home they could listen to with someone else. It’s a big thing for patients to come and hear all this and hear the word cancer, meet loads of people and it’s reassuring to know they’ve got it all with them somewhere when they get back [home].

C1

#### Communication and SecondEars

The most common opinion expressed by health professionals was that recording their consultations had not changed how they spoke or behaved toward patients, despite this being an initial concern. Instead, the health professionals felt that they could use SecondEars to identify areas where communication could be improved. It was also thought that having SecondEars in the appointment would encourage patients to ask more questions.

It can be difficult for patients to speak out and ask questions at the time and then if they don’t ask those questions, recalling their questions or recalling the prompt to those questions can be difficult. So for my groups specifically um knowing that they could listen to what was said and then come back to me with their questions was really good.

C1

Participating health professionals hypothesized that while they were comfortable with SecondEars, this may not be the case for all health professionals. They felt that some people may be uncomfortable with being audio-recorded, and that there may be some initial reluctance owing to concerns that lack of knowledge or poor communication skills could be *shown up*.

You know perhaps they feel a bit insecure in their clinical skills (laughs) or something and so they feel like being recorded might... could be used against them at some point or other or... I don’t know.

C9

Having a phone active in the consultation was discussed as potentially being a distraction or increased awareness of being recorded and so impact on establishing patient rapport (at least initially). However, participants reflected and felt that this had not occurred in practice during the study. It was also felt that recordings should not be used deliberately by either health professionals or patients to *prove* each other wrong about information discussed or missed within an appointment. Overall, it was thought that very few health professionals would be opposed to SecondEars being implemented, and that any initial discomfort regarding the app or recordings would diminish in time.

#### Practical Implementation Issues and Solutions

Important considerations regarding facilitation of use included how to ensure that interpreters were comfortable being recorded if non-English speaking patients were to download and use the app, or how to manage patients who do not own a smartphone or who struggle with using technology. Health professionals also raised process concerns, such as potential technical issues increasing already busy clinic times and the sustainability of the app. Data security was also discussed.

My other concern would be um if it adds time onto the consultation [for example] so if you’ve got 15 minutes to speak to your consultant about um... ah your illness it’s the first seven minutes of it wasted while you say oh where’s my phone, hang on my wife had it, it’s in a bag... you know you don’t want to lose that precious time with the consultant. They don’t want to lose that precious time with the patient

C1

One doctor also raised the potential for recordings to be shared inappropriately via social media; however, they themselves were comfortable with standing by whatever they had said in a consultation and felt that this was going to be a potential issue regardless of the patient using SecondEars to make a recording or not.

Literally any time you speak to a patient or any time you email somebody you have to... ah have an expectation that this could appear on Twitter or you know or the front page of the Herald Sun and am I prepared to stand over what you might have thought was, is still a confidential conversation but I think that’s just the way we... the society we live in.

C3

Solutions to these concerns were readily supplied. Health professionals suggested that written information about SecondEars be provided to patients upon admission or registration at the hospital, including detailed instructions on how to download and use the app. Hospital volunteers were proposed as appropriate persons to assist patients with app installation and use to reduce any technical issues and/or delays by ensuring that installation is completed before attending a consultation to. Information about SecondEars displayed prominently in clinic waiting rooms and consultation rooms to prompt patients planning to record to obtain consent from all persons in the room, reminders to start and stop recording, and details outlining patient responsibilities were suggested. Furthermore, several health professionals suggested using implementation strategies previously employed by other new technologies, such as telehealth or even other nontech strategies, such as health screening tools, as it was felt that these successful examples were implemented effectively and collaboratively. Finally, including communication skills training with implementation was seen as essential, as this would help mitigate health professionals’ concerns relating to communication, inappropriate sharing, or medico-legal issues.

So we could develop some pretty concise—not you have to go to a communications skills training workshop for the next weekend—but here’s some techniques that other people say are useful you know and then you can go OK I’m gonna try that so. It feels like there could be some training with the rollout.

C6

## Discussion

### Clinical Pilot Testing

To facilitate successful implementation, information about the functionality and suitability of new technologies is required. This study used patient, family, and health professional feedback to identify factors for optimal implementation.

Piloting SecondEars in a clinical setting was useful for determining how both patients and health professionals interacted with the app in conjunction with balancing their needs as a patient or role as a health professional. Importantly, not all patients chose to use SecondEars. This information is essential for planning appropriate infrastructure to support data management and storage of recorded files within the medical record. Although the app worked well for the majority of participants, some process issues were identified, such as issues with Wi-Fi or passwords. Implementation would require supportive frameworks and governance to ensure that users are able to access and use the app at the appropriate times, or that the app is adapted to address any identified barriers [36]. Most patient participants used the app to relisten to recordings or share with family and friends, validating the needs assessment, and mapping of use identified in the co-design process [17]. Integration of SecondEars into the current health care system is likely to be effective in overcoming some of the previous technological challenges seen with older consultation audio-recording research, as it has the potential to be sustainable, have strong data security, good sound quality, and low clinical burden [17].

### User Experience and Acceptability, Utility, and Satisfaction

Data from both the MARS and the patient interview provided a comprehensive indication of UX, and decisions or perspectives of acceptability, utility, and satisfaction of SecondEars. Comments about design and function focused on how simple the functions were, and how easy people found the app to use. Every participant confirmed that given the opportunity they would use SecondEars again, and recommend it to others, indicating that patients who want to audio-record consultations feel the app delivers this service well. Perceived usability, usefulness, and design quality have been identified as key criteria for uptake and continued use of technological innovations [23,30,36-38], and the results of this pilot suggest that SecondEars meets these criteria. Findings from this study underscore the benefits provided by involving end users in the design of mHealth solutions [38], as the final product aligns with patient needs and resulted in improved patient experience.

Moreover, SecondEars was specifically designed as a tool to improve communication, patient empowerment, and health care quality [17], while simultaneously overcoming one of the more significant barriers identified in previous consultation recording studies, sustainable facilitation, patient audio file provision, and storage of consultation recordings [16]. Our results suggest that SecondEars helps patients to feel empowered, and using their own phone to be in control of the audio-recording process gives them agency and flexibility. Health service interest in mHealth technologies is related to realigning service delivery to embody patient-centered care [5]. Health consumers likewise are looking to mHealth tools to assist with self-management of their care [7]. UX data from this pilot suggest that the app facilitates patient health care engagement and self-management, indicating that SecondEars will meet both health system and patient requirements.

### Implementation Strategy

Results from this pilot study suggest that health professionals support the implementation of SecondEars as an optional component of usual care. This indicates that mHealth is beginning to be accepted as a norm in routine health care, which will prove a positive driver for implementation [39]. For health professionals who participated in this study, this change in perspective extended to commonly discussed barriers to consultation recordings such as medico-legal concerns. Feedback from our sample reflected an attitude of acceptance, pragmatism, and focus on patient benefit, which deviated from the wariness that has been raised by health professionals in other studies [13,16,40]. Health professionals reported previous experience of both covert and permissive use of smartphones to record consultations, replicating previous research [11,41,42]. The ubiquity of smartphone use was seen as an additional driver for hospital culture change, and an argument for implementation to ensure the hospital has oversight of this process.

While our sample of health professionals may not reflect the majority of health care workers, reflections were made about other colleagues and peer perspectives regarding audio-recordings. Potential concerns were acknowledged, particularly that people may feel uncomfortable with being recorded. Possible unintended consequences recording consultations may have on doctor-patient communication emerged in this study, something which has been discussed at length in the literature [13,16,40,42]. However, again, the dialogue changed, as health professionals reported that anticipated negative side-effects of recording (disruption of rapport building) did not eventuate in practice, or if they did occur, they diminished rapidly with time, findings which have been supported by research demonstrating that recording does not affect clinical practice [43]. Furthermore, medico-legal and communication concerns were thought to stem from doubt or insecurity about communication skills. Several health professionals interviewed therefore recommended a communication skills training program to be designed and coimplemented with SecondEars to alleviate these barriers.

As noted above, both health professionals and patients volunteered to take part in this study. As per the CFIR framework, this is likely due to a complex interplay between the domains investigated (see Figure 1) such as characteristics of individuals, the intervention, inner settings of the organization (such as organization culture), and features of the intervention itself [30]. It could be postulated that our sample represents individuals who are *early adopters* of technology [44], and therefore, their perspectives are representative only of this group and not those who may be more wary or less enthusiastic about new technology in general, or of SecondEars in particular. An important aspect for future evaluation of integration of SecondEars into a clinical setting will be to understand in more detail which factors drive uptake and ongoing use of the app by patients and health professionals alike. While smartphone apps have been used specifically to improve health access and equity for disadvantaged populations [5,45], further investigation into whether SecondEars use improves health literacy or whether low health literacy is a barrier to use is needed.

Overwhelmingly, health professionals viewed SecondEars as a tool which enabled them to improve patient care and to improve the efficiency and quality of their work; 2 key facilitators for mHealth uptake noted in previous implementation studies [26,46]. As previously noted, new technologies are more likely to be taken up if they improve care delivery and do not impede or slow existing processes [36]. Implementation of SecondEars will therefore benefit from harnessing health professionals’ desire to provide the best possible patient care as a key driver for acceptance and uptake. Likewise, information regarding how the app can facilitate health professional role efficiency will also be useful. Health professionals in our study referred to implementation of other successful implementations of eHealth technologies, such as telehealth. Incorporation of implementation protocols which have already met with eHealth professional approval could also be a useful lever for acceptance.

### Limitations

Pilot testing was only conducted in 1 location, with a small sample of patients and health professionals. Pilot testing in health services other than a specialized oncology service would help identify and understand differing organizational processes which would require consideration for implementation. An evaluation of patterns of use with a larger sample of patients and health professionals would also provide more information regarding uptake, use, and those who do not elect to use the app. Furthermore, the SecondEars app was only available for testing in iOS and not in Android, which limited the sample of eligible patients. The mean age of participants (58 years) was slightly younger than the mean age of Australians at cancer diagnosis (66.3 years) [47]. It is possible that our younger sample may reflect a propensity for younger people to participate in mHealth research, however only 9% (2/23) of the patients who declined participation cited lack of confidence with technology as their reason for declining. In addition, the majority of participants were male, which was most likely a consequence of participating clinicians working in cancer specialties which typically have greater numbers of male patients (Urology, Gastrointestinal, and Lung). SecondEars is currently only available with English prompts; therefore, the study excluded people who could not speak English. Future versions of SecondEars are intended to include other languages to increase equity of access.

### Future Research

While clinical testing indicates positive responses from health professionals and patients alike, the next step will involve longer term implementation and evaluation of SecondEars. Integration of this solution within usual care would additionally provide a data collection platform to facilitate a range of additional research opportunities in doctor-patient communication, health literacy, and treatment/medication adherence.

### Conclusions

Data collected from pilot-testing SecondEars, in conjunction with patient and health professional perspectives, will be useful for developing a comprehensive strategy to implement SecondEars within a hospital setting. In particular, the app was met with support from the participants in this study and was seen by participants as useful in improving patient care and self-management and health service delivery. mHealth innovations which are most likely to succeed are those which focus on improved patient communication or supporting patient-centered care and those which improve patient empowerment and self-management [38,44]. Given the extensive research conducted on the benefits of consultation recordings [13,48,49], SecondEars as an mHealth solution has the potential to effectively deliver these benefits, and use patient and health professional experience to assist in developing a robust implementation strategy.

## Appendix

Multimedia Appendix 1 Screenshots of the SecondEars app design.

## Abbreviations

CFIR: Consolidated Framework for Implementation Research
eHealth: electronic health
MARS: Mobile App Rating Scale
mHealth: mobile health
Peter Mac: Peter MacCallum Cancer Centre
UX: user experience
